# Andersen health care utilization model: A survey on factors affecting the utilization of dental health services among school children

**DOI:** 10.1371/journal.pone.0286945

**Published:** 2023-06-15

**Authors:** Preethi Nagdev, Murali R. Iyer, Sachin Naik, Sanjeev Balappa Khanagar, Mohammed Awawdeh, Abdulaziz Abdullah Al Kheraif, Sukumaran Anil, Majed M. Alsarani, Sajith Vellappally, Omar Alsadon

**Affiliations:** 1 Department of Public Health Dentistry, SJM Dental College and Hospital, Chitradurga, India; 2 Department of Public Health Dentistry, Krishnadevaraya College of Dental Sciences, Bangalore, Karnataka, India; 3 Dental Biomaterials Research Chair, Dental Health Department, College of Applied Medical Sciences, King Saud University, Riyadh, Saudi Arabia; 4 Preventive Dental Science Department, College of Dentistry, King Saud Bin Abdulaziz University for Health Sciences, Riyadh, Saudi Arabia; 5 King Abdullah International Medical Research Centre, Ministry of National Guard Health Affairs, Riyadh, Saudi Arabia; 6 Department of Dentistry—Oral Health Institute, Hamad Medical Corporation, Doha, Qatar; 7 Dental Health Department, College of Applied Medical Sciences, King Saud University, Riyadh, Saudi Arabia; University of Catania: Universita degli Studi di Catania, ITALY

## Abstract

**Background:**

Children’s quality of life, academic performance, and future achievement can all be negatively affected by poor dental health. The present study aimed to assess the need for dental health services and the factors influencing their utilization using the Andersen health care utilization model among school children.

**Methods:**

The current cross-sectional study was conducted among schoolchildren aged 13 to 15 in Bangalore, India (n = 1100). A questionnaire was developed using the concepts of the Andersen healthcare usage model. The parents of the children filled out the questionnaire. The factors were investigated using bivariate analysis and multivariate logistic regression analysis.

**Results:**

About 78.1% of the children did not utilize dental health services. Regarding the reasons for not visiting a dentist, 65.8% said they did not have a dental problem, and 22.2% said they could not afford it. Bivariate analysis showed that age, gender, education level, occupation of the family’s head of household, monthly family income, socioeconomic status, perceived oral health problems, accessibility of dental health facilities, and parental attitudes toward their children’s oral health were significantly associated with using dental health services (p<0.05). Multiple regression analysis showed dental health service utilization was directly related to age (OR = 2.206), education, family size (OR = 1.33), and brushing frequency twice a day (OR = 1.575) with no significant relationship between distance to reach the dental facility, the number of dental visits, and socioeconomic status.

**Conclusion:**

Dental health service utilization was low in the past year. The age, number of family members, parent’s education level, travel time to the dental facility, the child’s oral health behaviors, and positive parental attitude all play a role in a children’s utilization of dental health service.

## Introduction

World Health Organisation (WHO) stated a 64–75% prevalence of dental caries among 15 year age children [1]. An early dental appointment provides appropriate preventive dental treatment and a good chance for dental health education. Pain, difficulty in eating, smiling, and communicating are symptoms of poor oral health. A discolored tooth and a missing tooth can affect a person’s daily life [2–4]. Children’s missing school because of oral problems is recognized as a public health and socioeconomic concern [5, 6]. Utilization of health services is the criterion for evaluating an individual’s healthcare utilization [7, 8].

Dental caries is the most critical indicator of oral health, while dental appointments are a marker of dental care [9, 10]. The school setting is thought to be the most efficient approach to reaching out to children’s families and communities [11].

Previous studies suggest that a family member should be psychologically prepared to respond to health dangers or circumstances [12, 13]. The number of dental clinic visits in a year is used as a standard measure to analyze dental health service(DHS) utilization [8]. Family income, parents’ education, employment, health insurance, parental preventative practice, behaviors, and access to dental care are the factors that influence the utilization of DHS [14]. Three factors that affect oral health care visits are dental anxiety, a preference for dental health protection, and a family dental health problem [15].

The healthcare system comprises of public and private healthcare providers. In many countries, the public healthcare system restricts the patient’s age or dental care service coverage [6, 7]. Most dental care procedures demand cost-sharing, or the patient entirely pays. Cross-national and even local authorities, there are significant differences in price amount and the kind of treatments that are not included in the benefits package [16, 17]. While dental care is an essential component of primary healthcare in India, it is only provided in a few states. Patients at both public and private dentists usually pay out of pocket because their insurance does not cover them. The utilization of DHS is low in areas with a sufficient supply of dental professionals, which widens the disparities in oral health between socioeconomic classes [18].

**Fig 1:**
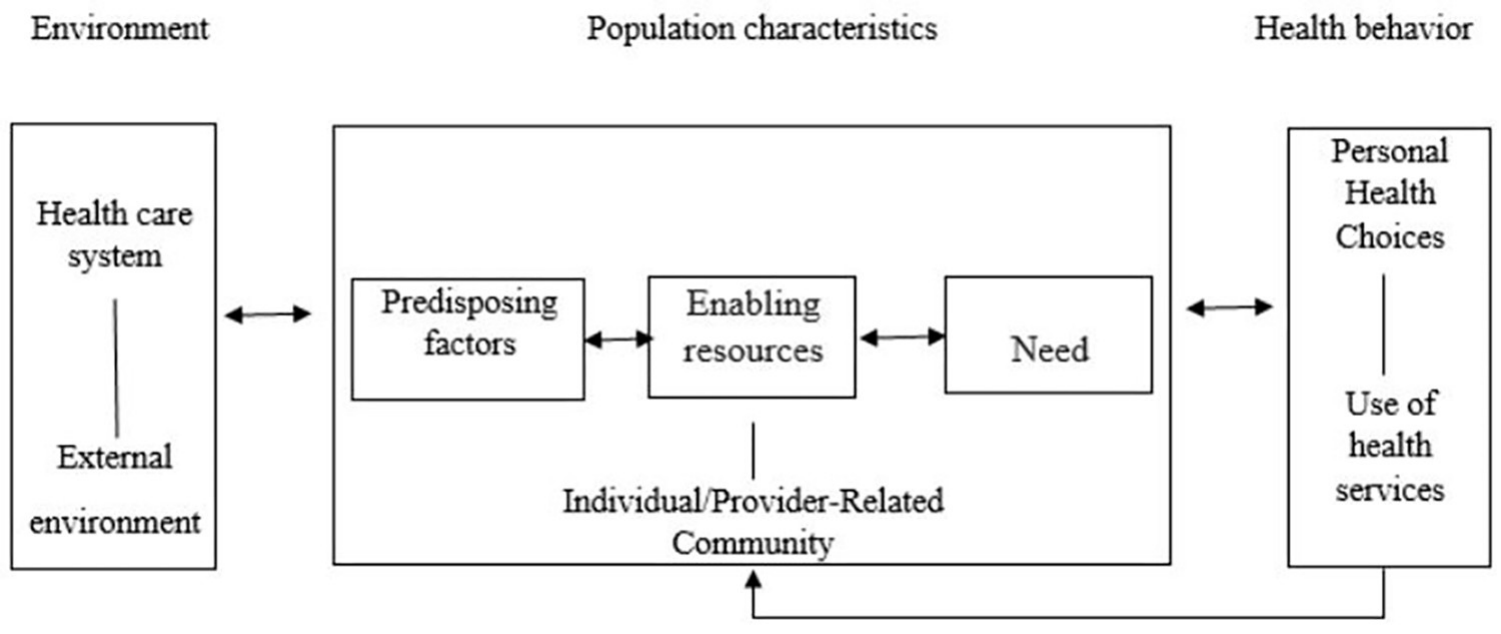
Andersen’s theoretical model.

In the late 1960s, Andersen’s health care utilization model (AHM) was developed to assess the health care services utilization by people. It combined the idea of how and why health services are used [19]. Many hypothetical healthcare utilization models have been presented based on behavioral, psychological, economic, and epidemiologic concepts [20–23]. The AHM is easier to use than other evaluation models. Andersen defines three key concepts explaining the use of health services that are: a) predisposing factors, b) enabling factors, and c) need-related factors (Fig 1).

Previous studies have used the AHM to assess DHS utilization [24, 25]. The model suggests a) predisposing factors such as children’s gender and psychosocial b) enabling factors such as socioeconomic status (SES), and c) need-related factors such as—Children’s DHS utilization and the number of dental visits. Children’s DHS utilization is predicted by parents/guardians’ attitudes [26, 27]. Using Andersen’s health care utilization model, our study aimed to analyze the factors influencing DHS utilization among 13 to 15-year age school children in Bangalore, India.

## Materials and methods

### Study area and period

The current cross-sectional study was conducted among schoolchildren aged 13 to 15 in Bangalore City, India. We surveyed to assess the utilization of DHS and the need for oral health services. The study was conducted over four months, from November 2014 to February 2015.

### Study population

Schoolchildren aged 13 to 15 years participated in the study. The parents of the children filled out the questionnaire.

### Eligibility

Parents or guardians who gave consent and children who showed up on the examination day were included in the study. Children who were mentally challenged or had systemic disorders were excluded from the study.

### Sample size and sampling technique

The sample size was calculated using the following formula:

$$(n)\;=\;\frac{Z^{2}\left(1-\frac{\alpha }{2}\right)(1-p)}{\varepsilon^{2\;}p}$$

n = Sample size

α = Significance level of a test (0.05)

Z = 1.96

ε = 0.05, variance estimated to be 5% (0.05)

p = 0.60 (p = dental caries, 60% of prevalence from the previous studies) [28].


$$(n)\;=\frac{\left(1.96^{2})(1-\frac{0.05}{2})\;(1-0.60\right)}{{(0.05}^{2})(0.60)}=\;998$$

The sample size obtained was 998. Considering a 10% deletion rate, we gathered a sample of n = 1100.

**Fig 2:**
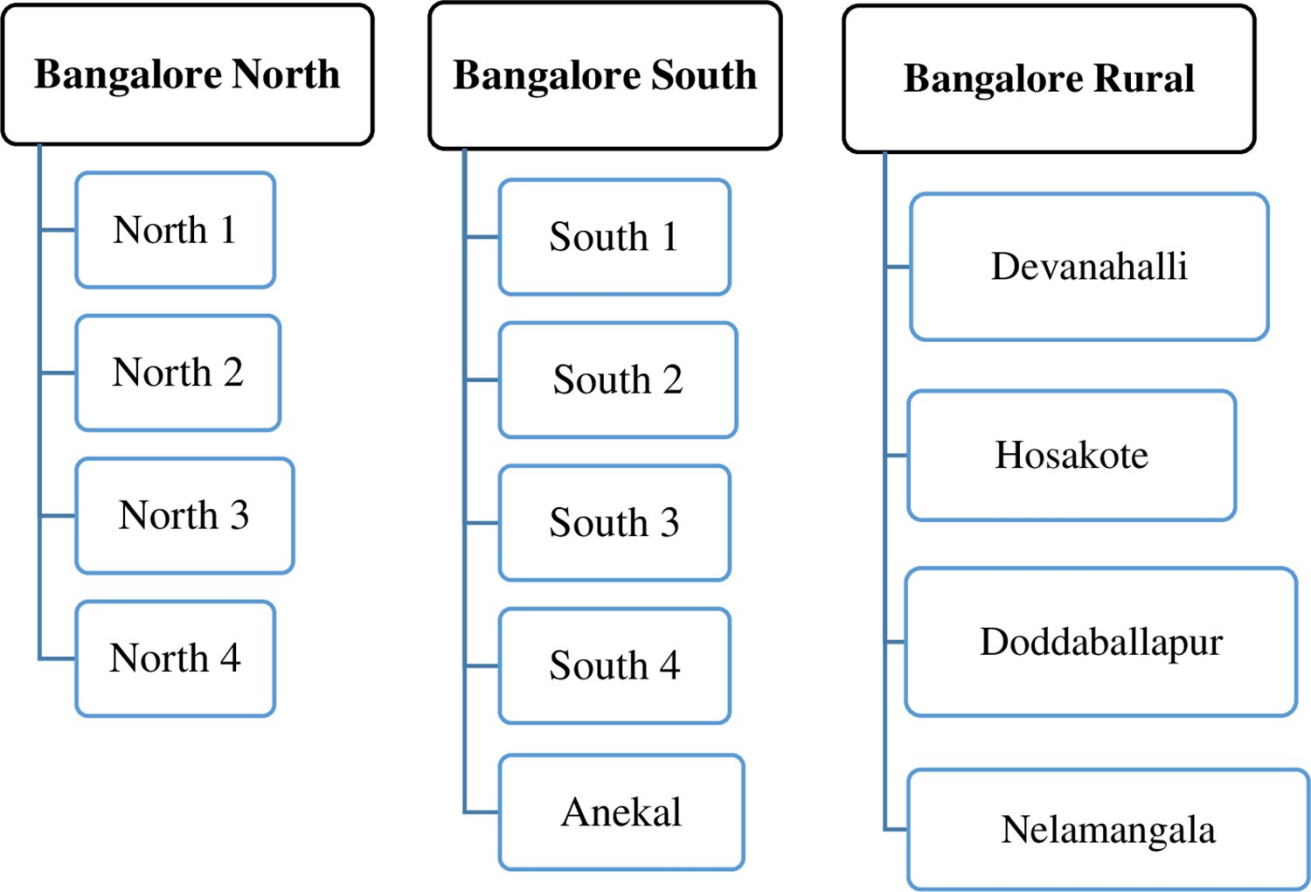
Flow chart indicating the distribution of schools in the Bangalore district.

Bangalore is the capital of Karnataka state. Bangalore district schools are separated into three zones for administrative purposes: Bangalore North, Bangalore South, and Bangalore Rural. Each zone is then further subdivided. The education board provided the list of schools. In the Bangalore North-4 subdivision, there were 201 high schools. For the study, a total of sixteen schools were chosen by simple random sampling (lottery method) (Fig 2).

In 201 schools, there were 31,907 students, 16,879 boys and 15,028 girls. On the day of the examination, study participants who satisfied the inclusion criteria were selected from each school using a simple random method by assigning a consecutive number and selecting randomly among them. The procedure was continued until the required sample was obtained. Finally, 1100 school-going children enrolled from 13 high schools across Bangalore North-4. **Data collection tool and procedure**

A self-administered questionnaire that follows the AHM’s guidelines was developed. The questionnaire comprised 19 closed-ended questions organized into five sections. The following were the contents of each section. Section 1–demographic and SES of the family, section 2–oral health service utilization by the child, section 3–availability of oral health services, section 4–child’s oral hygiene practices, and section 5–parental attitude towards the child’s oral health.

Before the questionnaire was used in the field, the content validity was evaluated by ten experienced local researchers, dental professors, and health administrators. The panel of experts agreed that the content validity was over 0.7.

To determine the feasibility of the study, 60 students from a school participated in a pilot study. After the pilot study, modifications were made to the questionnaire.

WHO oral health assessment proforma(2013) was used to record oral health status [29], and later, Decayed, Missing, and Filled Teeth (DMFT) index scores were derived from the proforma. Data on DHS utilization were collected using a specially built proforma based on the AHM [27]. SES comprises a composite score that considers the family head’s education and occupation and the family’s monthly income [30].

### Data quality control

To record the oral health status of children. Two examiners were trained and calibrated by the clinically competent senior faculty of the department to ensure uniform interpretation by the examiner for the various oral diseases and conditions to be observed for the proforma. For oral health status recording, the intra-examiner reliability of the two examiners was compared, and the Kappa value was found to be 0.87.

### Data analysis

From the collected data, frequency and percentages were calculated. The descriptive statistics of the key variables were reported. Chi-square analysis was used to assess the association between each variable and dental care visits.

Multiple logistic regression analysis was performed to determine the extent (OR) to which demographic, sociodemographic, and other variables influenced a dental visit in the previous year. The dependent variable was, whether the respondent had seen a dentist in the last year. The dentist’s visit in the previous year was chosen as the dependent variable because, in the Anderson model, other factors could affect this variable. The adjusted odds ratio (OR) and 95% confidence interval were determined using non-automated backward elimination, with the criterion for removal being 0.05 significance on the Chi-square test. Statistical significance was defined as p<0.05. SPSS version 22 was used for statistical analysis.

### Ethical consideration

Krishna Devaraya College of Dental Sciences and Hospital Bangalore provided ethical committee approval with the institutional review board (IRB) number RP/2013/229 dated 5^th^ November 2013.

## Results

### Sociodemographic variables

There were 391(35.5%) children of 13 years age, 437(39.7%) with 14 years and 272(34.7%) with 15 years. Male children were 582(52.9%) and females were 518(47.1%).

### Oral health status

Based on WHO proforma findings, four participants (0.4%) had enamel opacity/hypoplasia, 229 participants (20.8%) had dental fluorosis, 426 (38.7%) had decayed teeth, 18 (1.7%) had missing teeth, 39 (3.5%) filled teeth and mean decayed, missing, filled teeth (DMFT) score was 0.82 ±1.194. Among boys, 116 (19.9%) participants had healthy gum, 200 (34.4%) had bleeding gum, and 266 (45.7%) had calculus. Among girls, 111 (21.4%) participants had healthy gum, 183 (35.3%) had bleeding gum, 224 (43.3%) had calculus, and malocclusion was 48.3%.

### Dental health service utilization by children

The utilization of DHS according to age was 27% at 13 years of age, 45.2% at 14 years, and 27.8% at 15 years. Among them, 63.5% were males, and 36.5% were females.

Among study participants, 16.6% of the upper-lower-class participants used DHS compared to 54.4% of the upper-middle class. Regarding perceived oral health problems among children, 446(40.5%) participants had problems in the last year, and 654 participants (59.5%) had no problems.

The number of children who got their teeth extracted was 58 (5.3%), the number of children who got their teeth filled was 76 (6.9%), the number of children who had their teeth scaling done was 38 (3.5%), some children had general check-ups were 69 (6.3%), and 859 (78.1%) of the study participants did not receive treatment.

Children who had not visited the dentist because they did not have a dental problem were 556(65.8%), 52 (6.0%) said "No service available," 191 (22.2%) said "Cannot afford," 25 (2.9%) said "Afraid of the dentist," 21 (2.4%) said they were "Too busy," and 4 (0.4%) gave other reasons (Table 1).

**Table 1:** Details of the utilization of dental health service.

	N	(%)
**Child suffering from any teeth problem in the past 12 months**		
Yes	446	40.5
No	654	59.5
**Type of treatment received**		
Tooth removal	58	5.3
Tooth filling	76	6.9
Tooth cleaning	38	3.5
General check-up	69	6.3
No treatment received	859	78.1
**Reason for not visiting the dentist**		
Didn’t have a dental problem	566	65.8
No service available	52	6.0
Can’t afford	191	22.2
Afraid of dentist	25	2.9
Too busy	21	2.4
Any other reason	4	0.4
**Availability of dental facilities**		
Private practitioner/Private hospital	153	13.9
Government hospital	272	24.7
None	572	52.0
Don’t know	103	9.4
**Time taken to reach the dental facility with available transport**		
Less than half an hour	116	10.5
Half an hour to one hour	319	29.0
More than one hour	294	26.7
Can’t say	371	33.7
**How often does your child brush his/her teeth?**		
Once daily	664	60.4
Twice daily	433	39.4
After every meal	3	0.3
**How does your child generally clean his/her teeth?**		
Finger	18	1.6
Tooth brush	1082	98.4
**Parents examined their child’s oral cavity to find out they are healthy**		
Yes	696	63.3
No	404	36.7
**Parents thinking of taking their child to the dentist in the next 6 months**		
Yes	158	14.4
No	942	85.6

N = Number,% = percentage

### Availability of dental health services

The availability of DHS among the study population showed that 153 (13.9%) had access to a private dental hospital. The number of children who had access to government hospital services was 272 (24.7%), and the number of children who had no access to facilities was 572 (52%). About 103(9.4%) participants were unaware of dental service facilities in their residential area (Table 1).

When the parents were asked whether they had examined their child’s teeth to find out if they were healthy, 696 (63.3%) answered "Yes," and 404 (36.7%) answered "No (Table 1). The question assessed parents’ perception of oral health problems suffered by children. "Has your child suffered teeth problems in the past 12 months?" 446 (40.5%) participants parents answered "Yes," and 654 (59.5%) participants parents answered "No". Bivariate analysis revealed that age, gender, education level, occupation of the family’s head of household, monthly family income, socioeconomic status, perceived oral health problems, availability and accessibility of dental health facilities, as well as parental attitudes toward their children’s oral health, were found to be significantly associated with using dental health services (Table 2).

**Table 2:** Bivariate analysis of factors influencing the utilization of dental health services.

Variables	The child visited/consulted last year		χ2(p-value)
	Yes n (%)	No n (%)	
**Age**			
13 years	65(27.0)	326 (38.0)	9.913 (0.007*)
14 years	109 (45.2)	328 (38.2)	
15 years	67 (27.8)	205 (23.9)	
**Gender**			13.856(<0.001*)
Male	153 (63.5)	429 (48.9)	
Female	88 (36.5)	430 (50.1)	
**Education of the head of the household**			15.270(0.004*)
Illiterate	41 (17.0)	191 (22.2)	
Primary education	19 (7.9)	66 (7.7)	
Middle school education	82 (34.0)	229 (26.7)	
High school education	92 (38.2)	298 (34.7)	
Graduate	7 (2.9)	75 (8.7)	
**Occupation of the head of the household**			
Farmer	27 (11.2)	177 (20.6)	40.596(<0.001*)
Agriculture labour	30 (12.4)	116 (13.5)	
Business	50 (20.7)	153 (17.8)	
Profession	70 (29.0)	270 (31.4)	
White collar worker	0 (0)	23 (2.7)	
Skilled worker	60 (24.9)	99 (11.5)	
Unskilled worker	4 (1.7)	21 (2.4)	
**Monthly income of the family**			25.975(<0.001*)
Below Rs. 1600	0 (0)	20 (2.3)	
Rs. 1601–4809	14 (5.8)	72 (8.4)	
Rs. 4810–8009	41 (17.0)	196 (22.8)	
Rs. 8010–12019	97 (40.2)	250 (29.1)	
Rs. 12020–16019	68 (28.2)	193 (22.5)	
Rs. 16020–32049	21 (8.7)	124 (14.4)	
Above Rs. 32050	0 (0)	4 (0.5)	
**Socioeconomic Status**			29.390(<0.001*)
Upper class	0 (0)	15 (1.7)	
Upper middle class	70 (29.0)	286 (33.3)	
Lower middle class	131 (54.4)	316 (36.8)	
Upper lower class	40 (16.6)	242 (28.2)	
**Availability of DHS facilities**			45.828(<0.001*)
Private practitioner	54 (22.4)	99 (11.5)	
Govt. Hospital	55 (22.8)	217 (25.3)	
None	132 (54.8)	440 (51.2)	
Don’t know	0 (0)	103 (12.0)	
**Time taken to reach the dental facility with transport**			111.350(<0.001*)
Less than half an hour	35 (14.5)	81 (9.4)	
Half an hour to one hour	86 (35.7)	233 (27.1)	
Over one hour	105 (43.6)	189 (22.0)	
Can’t say	15 (6.2)	356 (41.4)	
**Have you ever examined your child’s teeth**			60.873(<0.001*)
Yes	204 (84.6)	492 (57.3)	
No	37 (15.4)	367 (42.7)	
**Thinking of taking the child to the dentist in the next 6 months**			132.502(<0.001*)
Yes	90 (37.3)	68 (7.9)	
No	151 (62.7)	791 (92.1)	

χ2 = Pearson chi-square value, * = statistically significant at p<0.05

DHS utilization was directly related to age, education, family size, and tooth brushing frequency in the multiple logistic regression model (Table 3), with no significant relationship between distance and the number of dental visits. The patient’s age significantly affected the frequency of dental visits (p<0.05). The age-related OR was 2.206, showing that people are more likely to visit the dentist as age increases. The number of family members was also a significant factor in determining how often a dentist was seen (p<0.05, OR = 1.33).

**Table 3:** Multiple logistic regression analysis for dependent variable–DHS utilization.

Variables	β	’p’-value	Odds Ratio(95% CI)
**Age**	0.791	<0.001*	2.206(1.666–2.922)
**Gender**	1.044	<0.001*	2.045(1.233–1.994)
**Occupation of the head of the household**			
Farmer			1(ref)
Agriculture labour	12.567	0.453	0.43(0.000–0.000)
Business	12.087	0.003*	1.45(0.000–0.000)
Profession	12.467	0.004*	1.23(0.000–0.000)
White collar worker	12.895	0.273	1.63(0.000–0.000)
Skilled worker	12.324	0.983	0.23(0.000–0.000)
Unskilled worker	12.785	0.342	1.76(0.000–0.000)
**Monthly income of the family**			
Below Rs. 1600			1(ref)
Rs. 1601–4809	0.32	0.472	1.505(0.000–0.000)
Rs. 4810–8009	0.66	0.965	0.254(0.000–0.000)
Rs. 8010–12019	0.46	0.003*	0.146(0.000–0.000)
Rs. 12020–16019	0.31	0.006*	0.338(0.000–0.000)
Rs. 16020–32049	0.78	1.368	0.254(0.000–0.000)
Above Rs. 32050	0.21	1.433	0.356(0.000–0.000)
**Family members**	0.285	<0.001*	1.330(1.203–1.471)
**Socio Economic Status**			
Upper class			1(ref)
Upper middle class	18.092	0.999	0.00(0.000–0.000)
Lower middle class	18.636	0.998	0.00(0.000–0.000)
Upper lower class	18.085	0.999	0.00(0.000–0.000)
**Education of the head of the household**			
Graduate			1(ref)
Illiterate	-1.084	0.121	0.338(-1.062–1.738)
Primary education	-1.924	0.004*	0.146(-1.178–1.47)
Middle school education	-1.280	0.033*	0.278(-0.922–1.478)
High school education	-1.082	0.051	0.339(-0.769–1.447)
**Availability of DHS facilities**			
Private practitioner			1(ref)
Govt. Hospital	18.087	0.996	0.00(0.000–0.000)
None	17.563	0.996	0.00(0.000–0.000)
Don’t know	18.044	0.996	0.00(0.000–0.000)
**Time taken to reach the dental facility**			
>one hour			1(ref)
< Half an hour	0.251	0.287	1.286(0.814–1.758)
Half an hour to one hour	0.409	0.020*	1.505(1.155–1.855)
Can’t say	0.376	0.034	1.409(1.455–1.466)
**Brushing frequency?**			
Once-daily			1(ref)
Twice daily,	0.454	0.018*	1.575(1.080–2.296)
After every meal	0.735	1.000	2.086(0.000–0.000)
**Parents examined their child’s oral cavity to ascertain they are healthy**			
Yes			1(ref)
No	-1.369	<0.001*	0.254(0.162–0.399)
**Thinking of taking your child to dentist in the next 6 months?**			
Yes			1(ref)
No	-2.009	<0.001*	0.134(0.083–0.218)

β = Regression coefficient, * = statistically significant at p<0.05, ref = reference level, CI-confidence interval

Parents’ education was a significant factor in deciding whether to visit a dentist. The OR for illiterates who had completed elementary education, middle school, and high school, respectively, were 0.338, 0.146, 0.278, and 0.339 (p<0.04). DHS was not significantly influencing dentist visits (p>0.05, OR = 0.996). There was no link between SES and DHS consumption (p>0.05, OR = 0.00). The time taken to reach the dental facility (p<0.05, OR = 1.505) was found to be a significant factor in deciding whether to go to the dentist.

Brushing teeth twice a day was seen as significant (p<0.05, OR = 1.575), whereas brushing teeth after each meal had a higher OR (OR = 2.086), showing a higher likelihood of seeing a dentist in the previous year. Parents thinking of taking their child to the dentist in the next six months was revealed to be a significant factor influencing the visit (p<0.05), and OR<1 shows they had a reduced chance of visiting a dentist in the previous year.

### Discussion

The utilization of health services is an indicator of a good working healthcare system. There is a significant disparity in using health services between different countries [31]. Recognizing the factors that influence dental service consumption can aid in overcoming potential obstacles and reducing oral health disparities. Our study found that utilization of dental health services among children was low in the last year. Children’s use of dental health services is influenced by a variety of factors, including their age, the size of their family, their parents’ education levels, how long it takes them to get to the dentist, how well they take care of their teeth, and their parents’ attitudes.

In our study, boys visited the dentist more than girls. According to Aqeeli A et al., 12.8% of girls reported routine dental check-ups, compared to 6.9% of boys [32]. In our study, most of the 14-year-old children had visited the dentist in the previous year for a routine dental check-up. A previous study was carried out among pre-schoolers in Beijing, China, to determine the trends in using oral health services and to define the factors that contributed to those trends. About 45.5% of children had used dental treatment in the previous year. Children with greater access to dental health treatments and whose parents or caregivers believed they had poorer oral health conditions were more likely to use these services [33]. A similar study was conducted on children attending government schools in Bangalore, India, to assess the use of oral health services from a parent’s viewpoint. The study result showed that in the previous 12 months, only 7.80% of the parents consulted a dentist clinic, and 62% had access to private dental care. Financial limitations (45.19%) and parents’ ignorance about the local dental clinics (21.84%) were the major obstacles to utilizing DHS [34]. According to a survey conducted in Mexico, the frequency of dental visits due to tooth pain was associated with higher dental needs. Boys who attended public schools were 70% more likely to have a dental visit than boys who went to private schools, and oral problems were one of their main reasons for attending. Girls who attended public schools used 28% more DHS than private institutions [35].

In the current study, most individuals fall into Kuppuswamy’s lower-middle-class SES categorization [30]. According to Bourdieu’s sociological theory, children with parents or guardians with higher levels of cultural, economic, and social capital would have better perceptions of oral health and the use of DHS [36]. A literature study found that 27.7% of low-income counties had similar outcomes. A previous study was conducted in Brazil using the AHM model. The study’s bivariate analysis showed that the youngest children from low-income families, those living in overcrowded homes with single parents, and those with low-educated parents had the highest rates of never seeing a dentist [37]. The parental socioeconomic status and educational level were reflected in the children’s dental care utilization.

In our study, general check-ups and dental fillings were common reasons for visiting the dentist, similar to the Denloye et al. [38] study found that fillings (50.8%) were the primary cause for utilization. Joycelin et al. [39] found that 8.7% of individuals used dental services for caries removal. According to Punamalli P et al. [40], most research participants (10.2%) underwent scaling treatment.

Multiple logistic regression analysis showed that age, the number of members in the family, education, time taken to reach the dental facility, tooth brushing frequency, and parental attitude towards a child’s oral health were significant factors affecting utilization [7, 41].

In this study, there was an inverse relationship between the time to arrive at the dental facility and the usage of dental services. A literature review by Han-A Cho et al. showed that patient preferences for using DHS were reflected in the travel time, which was proportional to the type of treatment received. This was especially true for expensive treatment procedures not covered by national health insurance. Across all geographies, the rural regions had the longest median travel time per incident [42]. But some previous studies revealed no relationship between the time to arrive at the dental facility and utilization [11, 43].

In our study, most children did not visit dentists because they had no dental problems. A previous study conducted in Nigeria showed that a lack of perceived need for dental care was the most common reason children did not seek treatment (64.3%). A total of 187 (93.5%) of the children surveyed said they think it is necessary to go to the dentist, and among those, 32.0% said they would go if they were in pain [39]. These children’s low rates of dental care utilization are mainly attributable to a lack of awareness of the perceived need. There is a need to raise children’s consciousness about the value of routine dental check-ups for good oral health.

The study participants second most common reason for not using dental treatments was "cannot afford," showing that cost was one of the most critical determinants in utilization. A few of them also stated that "no service available" and "fear of the dentist" were reasons for not using dental health services in the previous year. According to A G Harikiran et al., 46.1% of the study participants, dental fear was the primary motivator for irregular visits [44].

To avoid or control tooth decay, parents have a crucial role in starting and reinforcing behaviors linked to oral health, such as cleaning teeth twice. Parents must encourage children to attend the dentist regularly and install good eating and oral hygiene habits. In our study, most parents were willing to take their children to dentists. According to study results conducted in India, parents had a favourable attitude and view of how much time, money, and dental appointments are spent on their child’s oral health [45]. According to a study conducted in Saudi Arabia, parents were more aware of preventative dentistry, but their use was closely connected with their level of education and money [46].

### Study limitations and strengths

The current study is a cross-sectional study. As a result, there is a built-in constraint of temporality. Data was gathered via a self-administered questionnaire, so the results may be inaccurate. Higher percentages of incomplete questionnaires limit self-reported data; however, this was not seen in the current investigation. It could not distinguish whether a visit was conducted in response to an emergency or a necessity. As a result, a visit to the dentist in the previous year could show either good preventative practice or an acute condition for the respondent. Dental insurance coverage, a determinant of the utilization of DHS, is in its infancy in India. Because of this, data was not collected on this aspect. The results can be generalized as a sample of participants represents the population, and a more significant number of response samples are collected.

### Conclusion

According to Andersen’s model, the child’s age, the number of family members, education, the time required to access a dental service, and a positive parental attitude toward a child’s oral health influence dental health service utilization. Most of the children needed dental health services, but they were underutilized. The majority of study participants underwent restoration treatment. Most schoolchildren had oral diseases such as dental caries, periodontal disease, and malocclusion. Programs that promote oral health and the availability of public hospitals are necessary to boost the use of dental services, improve parental and children’s attitudes toward them, make them more accessible and affordable, and remove any remaining hurdles.

## Supporting information

S1 Dataset (XLSX)

S1 File Questionnaire for utilization of oral health services. (DOCX)
