# Supplementary material for: Andersen health care utilization model: A survey on factors affecting the utilization of dental health services among school children
Source: PLoS One. 2023 Jun 15;18(6):e0286945. doi: 10.1371/journal.pone.0286945 (PMC10270576; doi:10.1371/journal.pone.0286945)
Supplement: S1 File — (DOCX) [file pone.0286945.s002.docx]

KRISHNADEVARAYA COLLEGE OF DENTAL SCIENCES AND HOSPITAL

DEPARTMENT OF PUBLIC HEALTH DENTISTRY

QUESTIONNAIRE FOR UTILIZATION OF ORAL HEALTH SERVICES

Name: Age: Sex: Date:

I. Questions to child’s parent/guardian on socioeconomic status of the family.

Name of head of the household

1. How many members are there in your family?
2. What is the level of education attained by head of the household?
3. Illiterate
4. Primary school
5. Middle school
6. High school
7. Graduate
8. Professional

03. Household’s occupation or profession

- 1. Farmer
  2. Agriculture labour
  3. Business
  4. Profession
  5. White collar worker
  6. Skilled worker
  7. Unskilled worker
  8. Others (Specify)

04. How much is monthly income of your family?

1. Below 1,000
2. 1,001-2,99
3. 3,000-4,999
4. 5,000-7,499
5. 7,500-9,999
6. 10,000-19,999
7. Above 20,000

II. ON ORAL HEALTH SERVICE UTILIZATION BY CHILD.

05. Has your child suffered from any teeth problems in the past 12 months?

1. Yes
2. No
3. Not sure

06. If yes, what was the problem?

- 1. Tooth decay
  2. Gum disease
  3. Broken tooth
  4. Bad smell
  5. Others (Specify)
  6. Not applicable

07. If no, mention the reason for not visiting the dentist.

- 1. Do not have a dental problem
  2. No service available
  3. can’t afford
  4. Afraid of dentist
  5. Too busy
  6. Others (Specify)
  7. Not applicable

08. What was the number of dental visits during the past 12 months?

- 1. Once in a year
  2. Twice in a year
  3. More than two times in a year
  4. Not visited

09. What kind of treatment your child has received?

- 1. Teeth removal
  2. Filling
  3. Cleanning
  4. General check up
  5. Others (Specify)
  6. None

10. How was the care at the last dental visit?

1. Good
2. Bad
3. Not applicable

III. ON AVAILABILITY OF ORAL HEALTH SERVICES.

11. What are the available dental facilities in your area?

1. Private practitioner
2. Govt. hospital
3. None
4. Don’t know

12. What is the time taken to reach the dental facility with available transport?

1. Less than half an hour
2. Half an hour to one hour
3. More than one hour
4. Can’t say

IV. ON ORAL HYGIENE PRACTICES.

13. How often does your child brushes his/her teeth?

1. Once daily
2. Twice daily
3. After every meal
4. Don’t clean every day

14. How does your child generally clean his/her teeth?

1. Finger
2. Tooth brush
3. Chewing stick
4. Others (Specify)

V. ON PARENTAL ATTITUDE TOWARDS CHILD’S ORAL HEALTH.

15. Your child’s dental health status?

1. Excellent
2. Good
3. Fair
4. Poor

16. Have you ever examined his/her teeth to ascertain if they are healthy?

- 1. Yes
  2. No

17. Does your child currently need any dental services?

1. Yes
2. No

18. Are you thinking of taking your child to see a dentist in the next 6 months?

1. Yes
2. No
